# Short‐Course Low‐Dose Primaquine for Radical Cure in G6PD‐Normal Patients in the Pre‐Elimination Context of Nepal

**DOI:** 10.1111/tmi.70145

**Published:** 2026-04-25

**Authors:** Prakash Ghimire, Gokarna Dahal, Nabaraj Adhikari, Komal Raj Rijal, Sanjib Adhikari, Megha Raj Banjara, Hellen Mnjala, Grant Lee, Sophie Weston, Angela Rumaseb, Anjana Rai, Benedikt Ley, Mohammad Sharif Hossain, Julie A. Simpson, Megha Rajasekhar, Bipin Adhikari, Ric N. Price, Bibek K. Lal, Kamala Thriemer

**Affiliations:** ^1^ Central Department of Microbiology Tribhuvan University Kirtipur Kathmandu Nepal; ^2^ National Malaria Program, Epidemiology and Disease Control Division, Department of Health Services Ministry of Health & Population, Government of Nepal Kathmandu Nepal; ^3^ Global and Tropical Health Division, Menzies School of Health Research Charles Darwin University Darwin Northern Territory Australia; ^4^ Infectious Disease Division International Centre for Diarrhoeal Disease Research Dhaka Bangladesh; ^5^ Centre for Epidemiology and Biostatistics, Melbourne School of Population and Global Health University of Melbourne Melbourne Victoria Australia; ^6^ Centre for Tropical Medicine and Global Health, Nuffield Department of Medicine University of Oxford Oxford UK; ^7^ Mahidol Oxford Tropical Medicine Research Unit Bangkok Thailand; ^8^ Family Welfare Division, Department of Health Services Ministry of Health & Population, Government of Nepal Kathmandu Nepal

**Keywords:** G6PD, Nepal, radical cure, randomised trial, short‐course low‐dose primaquine, vivax malaria

## Abstract

**Background:**

*Plasmodium vivax* remains a challenge for malaria elimination in Nepal due to its ability to relapse. Radical cure with primaquine is effective but limited by poor adherence to the standard 14‐day low‐dose regimen. In 2022, the WHO recommended administering the same total dose (3.5 mg/kg) over 7 days to improve adherence. This study aimed to evaluate the 7‐day low‐dose primaquine regimen in G6PD‐normal patients with uncomplicated *P. vivax* and/or *P. falciparum* malaria in the pre‐elimination context of Nepal.

**Methods:**

A randomised study was conducted in south‐west Nepal. Adult patients with microscopically confirmed *P. vivax* and/or *P. falciparum* malaria and glucose‐6‐phosphate dehydrogenase (G6PD) activity ≥ 30% were randomised 1:1 to receive either a 7‐day low‐dose primaquine regimen (0.5 mg/kg/day; total 3.5 mg/kg) or standard of care (14‐day primaquine [0.25 mg/kg/day; total 3.5 mg/kg] for patients with *P. vivax* malaria and single‐dose primaquine for *P. falciparum* malaria patients) in addition to their schizonticidal treatment. All treatment was directly observed, and patients were followed for 6 months. The primary outcome was the risk of *P. vivax* recurrence at 6 months. Safety outcomes included adverse events, gastrointestinal symptoms and haematological parameters.

**Results:**

Of 5842 individuals screened, 27 eligible participants were enrolled. Among these, 21 had *P. vivax* , four had *P. falciparum* and two had mixed infections. Eleven participants had intermediate G6PD activity (≥ 30%–70% activity). At 6 months there were no recurrences in the 7‐day primaquine arm (*n* = 14) and the risk of *P. vivax* recurrence in the standard primaquine arm (*n* = 13) was 9.1% (95% CI: 1.3–49.2). No participants vomited study drugs, and gastrointestinal symptoms were infrequent. Three participants experienced a ≥ 25% haemoglobin drop between baseline and Day 2, all of whom had baseline values > 15 g/dL. No presentations of haemoglobinuria, severe anaemia, serious adverse events, or deaths occurred during the study period.

**Conclusions:**

In this small study, the 7‐day low‐dose primaquine regimen was well tolerated, including among individuals with intermediate G6PD activity. Although the small sample size limits conclusions about efficacy, the findings support the feasibility and safety of this regimen in Nepal, offering potential programmatic advantages for radical cure delivery in a setting close to elimination.

**Trial Registration:**

Clinicaltrials.gov: NCT04079621

## 1 Background

Over the past two decades, Nepal has achieved remarkable progress in reducing the burden of malaria with a 92% reduction in the overall malaria burden between 2002 and 2016 [1]. As Nepal moves closer to its national elimination target, vivax malaria has emerged as the principal obstacle to achieving and sustaining elimination.

Unlike *Plasmodium falciparum*, *Plasmodium vivax* forms dormant liver‐stages (hypnozoites) that can reactivate weeks to months after an initial infection. Radical cure—the treatment of both blood‐ and liver‐stage parasites—is therefore essential. The only currently available drugs capable of killing hypnozoites are the 8‐aminoquinolines primaquine and tafenoquine. Most endemic countries use a low‐dose primaquine regimen (3.5 mg/kg total dose) administered over 14 days due to concerns over haemolysis in patients with glucose‐6‐phosphate dehydrogenase (G6PD) deficiency. However, adherence to this prolonged regimen is often poor, limiting its effectiveness [2, 3]. To improve adherence, the WHO updated their guidelines in 2022 to recommend that the low‐dose primaquine regimen could be administered over 7 days at a higher daily dose (0.5 mg/kg/day) [4]. This 7‐day regimen has been used in South America for several decades. Following the release of the revised WHO guidelines, several countries in the Asia Pacific, including Laos, Cambodia and Pakistan, updated their malaria treatment guidelines to include the shorter primaquine regimen [5, 6].

More recently, an individual patient data pooled meta‐analysis suggested that a higher total dose of primaquine is needed for adequate anti‐relapse efficacy [7]. Increasing the total dose of primaquine to 7 mg/kg almost halves the risk of recurrent malaria and this is apparent in nearly all *P. vivax* endemic areas [7]. The 2024 WHO guidelines revisions therefore support the use of higher‐dose regimens [8]. However, patients with *P. vivax* in South Asia have a lower risk of relapses compared to other endemic areas. A meta‐analysis of 791 individual patient records from South Asia, including data from Nepal, found a minimal reduction in relapses when comparing the 3.5 mg/kg dose to the higher 7 mg/kg regimen [9]. However, data from South Asia are limited, and few clinical studies with sufficient follow‐up have been conducted assessing the comparative efficacy and safety of different primaquine dosing regimens in this region [9].

In regions co‐endemic for *P. falciparum* and *P. vivax* , patients treated for *P. falciparum* malaria have an increased risk of subsequent *P. vivax* parasitemia. In a large meta‐analysis, 13%–42% of patients presenting with *P. falciparum* malaria developed *P. vivax* parasitemia within 2 months, believed to be due to reactivation of hypnozoites [10]. These findings support a universal radical cure strategy, where hypnozoitocidal treatment is administered to all patients with uncomplicated malaria, regardless of *Plasmodium* species. Prospective clinical trials have demonstrated that radical cure administered to patients with *P. falciparum* reduces the risk of subsequent *P. vivax* by fivefold in a multi‐centre trial [11] and threefold in a cluster randomised trial in Indonesia [12]. Although treating all patients with uncomplicated malaria with primaquine radical cure has pragmatic advantages, the benefits may be less apparent in areas of low malaria transmission. In a longitudinal analysis on the Thai‐Myanmar border, the risk of *P. vivax* recurrence following *P. falciparum* infections declined as the overall incidence of malaria fell [13].

In a pre‐elimination setting such as Nepal, opportunities to generate clinical evidence are inherently limited. Nevertheless, carefully documented studies are critical to inform national policy and contribute to global understanding of vivax elimination strategies. Against this backdrop, this clinical study aimed to assess the safety and efficacy of a short course primaquine regimen (3.5 mg/kg total dose administered over 7 days) compared to standard primaquine treatment in patients with G6PD activity ≥ 30% presenting with uncomplicated malaria due to any species and provide an operationally feasible option for radical cure in the context of malaria elimination.

## 2 Methods

### 2.1 Study Context

The study period overlapped with the COVID‐19 pandemic, during which country‐imposed lockdowns intermittently paused community‐based malaria activities and outreach, and malaria programme delivery was disrupted including reduced testing and operational constraints, while malaria diagnosis and treatment through routine services continued to be encouraged [14]. Study sites included primary care hospitals in Malakheti, Mahakali and Tikapur in the far western region of Nepal (Sudurpaschim province) bordering India. National data from 2021/22, 2022/23 and 2023/24 showed that the total number of malaria cases rose from 491, 533, to 791, with *P. vivax* responsible for 377 (76.8%), 382 (71.7%), and 618 (78.1%) cases respectively [1]. Provincial surveillance at the study sites reported a total of 198 malaria cases in Sudurpaschim province in 2023 of which 168 (84.8%) were *P. vivax* malaria with only six (3.0%) indigenous cases [1].

### 2.2 Study Design

This study was a randomised, open‐label, pilot study. The aim of the study was to generate efficacy and safety data from patients presenting with *P. vivax* or *P. falciparum* malaria treated with either 7‐day low‐dose primaquine regimen (0.5 mg/kg/day, total dose 3.5 mg/kg) in or the current standard of care (14 day low‐dose primaquine; 0.25 mg/kg/day, total dose 3.mg/kg for *P. vivax* patients and single dose primaquine for *P. falciparum* patients).

### 2.3 Participants

Eligible patients were 18 years of age or older with a confirmed diagnosis of *P. vivax* and/or *P. falciparum* malaria, as determined by light microscopy or a rapid diagnostic test (RDT) and fever (axillary temperature ≥ 37.5C) or history of fever in the preceding 48 h. Patients were excluded if they were pregnant or lactating, G6PD deficient (< 30% activity equivalent to 4 U/gHb) as measured by STANDARD G6PD (SD Biosensor, Gyeonggi‐do, South Korea), had anaemia defined as haemoglobin < 8 g/dL measured by STANDARD G6PD, had severe malaria requiring hospitalisation, had known hypersensitivity to any of the study drugs, used of other drugs with haemolytic potential, or had a blood transfusion in the last 4 months. Patients from mobile and migrant populations were excluded as they were unlikely to attend follow‐up visits and their inclusion would result in significant attrition bias. Written informed consent was obtained from all participants prior to enrolment.

### 2.4 Randomisation

Participants were randomised in a 1:1 ratio to either the 7‐day (intervention) or standard of care primaquine arm using a computer‐generated randomisation sequence. Sealed, opaque and sequentially numbered envelopes containing allocations were prepared by an independent team member before study start. Individual envelopes were only opened upon enrolment, thereby allocating patients to a treatment group.

### 2.5 Intervention

Participants were assigned to treatment groups based on their diagnosis of malaria and randomised to receive either a 7‐day primaquine regimen or standard care primaquine regimen.

All patients diagnosed with *P. vivax* malaria were treated with chloroquine (CQ) at a total dose of 25 mg/kg over 3 days, in accordance with national treatment guidelines, to clear the blood‐stage infection. Patients with *P. vivax* malaria randomised to the intervention arm were prescribed primaquine radical cure at a dose of 3.5 mg/kg, administered at 0.5 mg/kg/day over 7 days. Patients with *P. vivax* randomised to the control arm were treated with a total 3.5 mg/kg dose of primaquine administered over 14 days (0.25 mg/kg/day) as per national guidelines.

All patients diagnosed with *P. falciparum* malaria or mixed infection were treated with artemether‐lumefantrine (AL), as per national malaria treatment guidelines and those randomised to the intervention arm were treated with primaquine (total dose 3.5 mg/kg, 0.5 mg/kg/day for 7 days). Patients randomised to the control arm received a single dose of primaquine (0.25 mg/kg) for transmission‐blocking purposes, as per national guidelines. All treatment was directly observed by the study team, and any missed doses were recorded.

### 2.6 Procedures

Data were collected according to a structured schedule based on pre‐defined study visits. At screening and enrolment (Day 0), demographic data, medical history and baseline clinical assessments were recorded. A venous or capillary blood sample was collected for blood film examination, haemoglobin measurement and G6PD testing (STANDARD G6PD). Quality control was performed using STANDARD G6PD Control (Level 1 and Level 2), and analyser function was verified using the STANDARD G6PD Check Strip (SD Biosensor). Blood film examination was done per 200 white blood cells at the time of patient visit. All enrolment slides and a random subset of follow‐up slides were confirmed at trial completion by a WHO certified (Level 1 competency) expert microscopists.

All women of childbearing age underwent a urine β‐HCG pregnancy test to confirm eligibility.

Follow‐up visits were scheduled on days 1, 2, 3, 7, 14, 21, 28, and at months 2, 3, 4, 5 and 6. At each visit, clinical assessments included symptom evaluation, physical examination and capillary blood taken for haemoglobin measurement (STANDARD G6PD) and blood film examination. Patients were instructed to return to the clinic at any time if they developed febrile illness or symptoms suggestive of malaria. Adverse events, including haemolysis‐related complications, were assessed at each visit.

### 2.7 Outcomes

The primary outcome of the study was the recurrence of *P. vivax* parasitemia within 6 months, confirmed by microscopy. Secondary efficacy outcomes included the risk of symptomatic *P. vivax* malaria at Day 63, the risk of *P. vivax* malaria at 6 months in patients initially enrolled with *P. vivax* and the risk of *P. vivax* malaria at 6 months in patients initially enrolled with *P. falciparum*. Additional secondary outcomes included the incidence risks of *P. vivax* malaria at Days 28 and 42 among all patients.

Safety outcomes included the proportion of patients vomiting their medication within 1 h of administration, the proportion of patients vomiting any dose of primaquine during the supervised course, and the proportion of adverse events and serious adverse events reported within the first 7 days and before day 42. Gastrointestinal events were reported based on the symptom checklist and reported as a composite including nausea, vomiting and abdominal discomfort.

Haematological safety endpoints included the incidence risk of severe anaemia (Hb < 5 g/dL) or moderately severe anaemia (Hb < 7 g/dL) and/or the need for blood transfusion within 6 months, the incidence risk of a ≥ 25% fall in haemoglobin from baseline at Days 2 and 7, with and without haemoglobinuria, and the incidence risk of an acute drop in haemoglobin of > 5 g/dL during primaquine treatment.

### 2.8 Statistical Analysis

This study was designed as a pilot study not providing sufficient power to allow a non‐inferiority comparison. Therefore, no formal sample size calculation was done. It was expected that up to 100 patients would be recruited within 1 year; however, this target was not reached and the final sample size of 27 precluded formal statistical comparison.

The efficacy analysis was done on the principle of intention‐to‐treat, comparing the cumulative incidence of *P. vivax* parasitemia (time to first recurrence) between Day 14 and Month 6 (or Day 28 or 42 for secondary outcomes) estimated by Kaplan–Meier analysis, in the control (standard care) and intervention (7‐day primaquine) arms. For the primary outcome analysis, patients were censored at the day before a gap in blood film examination that exceeded 63 days.

The percentage of patients experiencing safety outcomes was calculated as the number of patients with the outcome divided by the number of patients for whom a response to the presence of the symptom or haemoglobin measurement was available at the given time point. Analyses were conducted using Stata v18.0.

### 2.9 Ethics

The study was approved by the Human Research Ethics Committee of the Northern Territory Health and Menzies School of Health Research (Ref # 19‐3467) and the Nepal Health Research Council or the Government of Nepal (Ref # 857/2019). All patients provided written informed consent before recruitment into the study.

## 3 Results

### 3.1 Study Population

**FIGURE 1:**
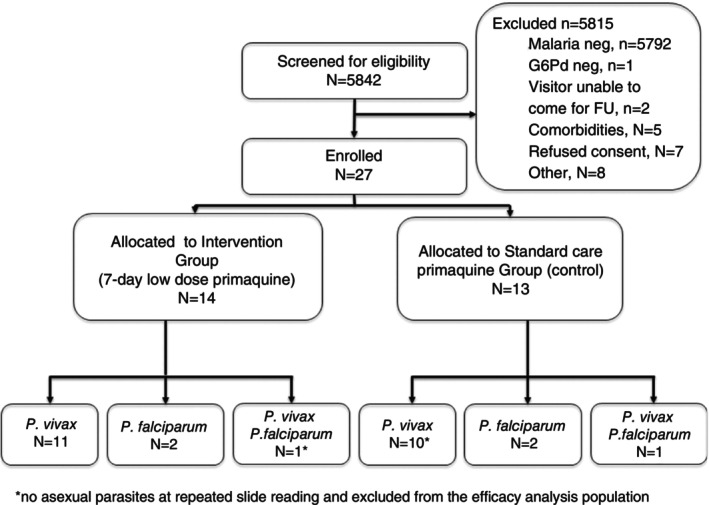
Allocation of patients in the study.

Between August 2021 and August 2023, a total of 5842 participants were screened for eligibility of whom 5815 (99.5%) were excluded predominantly because they did not have malaria (Figure 1). A total of 27 participants were enrolled in the study; 14 were randomised to the intervention group (7‐day primaquine) and 13 to the control group. In total, 21 were diagnosed with *P. vivax* malaria, four with *P. falciparum* malaria and two with a mixed infection. One patient enrolled with a mixed infection and allocated to the intervention arm was subsequently found to have no asexual parasites on repeated slide reading and was excluded from the efficacy analysis but included in the safety analysis. Of the 21 patients with *P. vivax* mono‐infection, 11 were randomised to the intervention arm and 10 to the control arm (standard care primaquine). Of the four patients with *P. falciparum* infection, two were assigned to each treatment arm.

The baseline characteristics of all enrolled participants are summarised in Table 1. The median age of patients in the intervention arm was 31 years (interquartile range [IQR] 22–51) and in the control arm was 22 years (IQR 19–41). Overall, 23 patients (85.2%) were male. A total of 11 (42%) patients had G6PD enzyme activities in the intermediate range (4–6 U/gHb). At baseline, the mean haemoglobin concentration was 15.7 g/dL (SD 2.0) in the intervention arm and 14.8 g/dL (SD 2.8) in the control arm. Fever at enrolment was observed in 24 of the 27 (89%) participants.

**TABLE 1:** Baseline characteristics by primaquine treatment group.

Characteristic	Intervention Group (7‐day low‐dose primaquine)^a^ (*n* = 14)^c^	Standard care primaquine group^b^ (*n* = 13)
Male	12 (86%)	11 (85%)
Median age (years)	31 (22–51)	22 (19–41)
Median weight (kg)	63 (53–69)	57 (54–62)
*P. vivax*	11 (78%)	10 (77%)
*P. falciparum*	2 (14%)	2 (15%)
Mixed infection	1 (7%)	1 (7%)
Fever (≥ 37.5C axillary)	12 (86%)	12 (92%)
Median G6PD activity (U/gHb)	6.1 (5.2–6.8)	6.3 (5.5–7.6)
G6PD normal (> 6 U/gHb)	7 (50%)	7 (54%)
G6PD intermediate (> 4–6 U/gHb)	5 (36%)	6 (46%)
Mean Haemoglobin (g/dL)	15.7 (2.0)	14.8 (2.8)

*Note:* Data are presented as mean (SD) or median (IQR) for continuous measures, and *n*/total (%) for categorical measures.

^a^ Patients with *P. vivax* malaria were treated with chloroquine and primaquine (total dose 3.5 mg/kg, 0.5 mg/kg/day for 7 days). Patients with *P. falciparum* malaria were treated with artemether‐lumefantrine and primaquine (total dose 3.5 mg/kg, 0.5 mg/kg/day for 7 days).

^b^ Patients with *P. vivax* were treated with chloroquine and primaquine (total dose 3.5 mg/kg, 0.25 mg/kg/day for 14 days). Patients with *P. falciparum* malaria were treated with artemether‐lumefantrine and a single dose of primaquine (0.25 mg/kg) for transmission‐blocking purposes.

^c^ One patient with mixed infection had no asexual parasites at repeated slide reading and was excluded from the efficacy analysis population, but not the safety analysis population.

Study treatment was fully supervised, and no doses were missed. A total of five (18.5%) patients (four in the intervention arm and one in the control arm) were lost to follow‐up before their final visit on Month 6. All patients lost to follow‐up were male and had *P. vivax* parasitaemia at enrolment, with no other notable demographic differences between them and patients followed up until Day 180.

### 3.2 Efficacy

**FIGURE 2:**
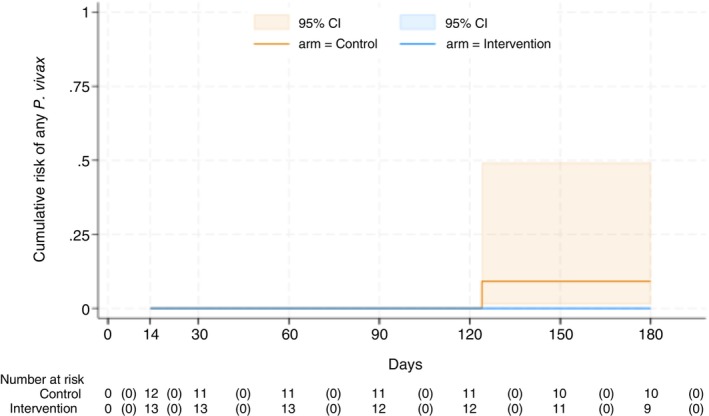
Cumulative incidence of any *P. vivax* within 6 months. Standard care primaquine for patients with *P. vivax* was 3.5 mg/kg total dose, 0.25 mg/kg/day for 14 days and for patients with *P. falciparum* it was a single dose of primaquine (0.25 mg/kg) for transmission‐blocking.

At 6 months, there was one episode of *P. vivax* parasitemia which occurred on Day 124 in a patient in the control arm initially presenting with *P. falciparum* malaria. The overall risk of *P. vivax* recurrence at 6 months was 0% in the intervention arm and 9.1% (95% CI: 1.3–49.2) in the control arm (Figure 2). There were no recurrences by Day 28 or 42 in either treatment arm.

### 3.3 Safety

A total of four patients reported vomiting in the previous 24 h on Day 0, but no patients vomited any of the study drugs within 1 h of administration. Based on a symptom checklist, gastrointestinal‐related symptoms on Day 3 were reported by 7.7% (1/13) in the intervention arm and 0.0% (0/13) in the control arm. There were no adverse or serious adverse events reported, and no deaths occurred during the study.

Three participants (23.1%) experienced a ≥ 25% drop in haemoglobin between baseline and Day 2, 15.4% (2/13) in the intervention arm and 7.7% (1/13) in the control arm. All three patients had high starting haemoglobin measurements greater than 15 g/dL and none had signs of clinical compromise. No patients reported haemoglobinuria (Table 2) or developed moderate (Hb < 7 g/dL) or severe (Hb < 5 g/dL) anaemia. None of the patients had a decrease in haemoglobin of 5 g/dL or greater.

**TABLE 2:** Details of the three patients with ≥ 25% haemoglobin drop between baseline and Day 2.

Study arm	Age	Sex	G6PD activity at enrolment (U/g Hb)	Haemoglobin (g/dL)			
				Enrolment	Day 2	Day 7	Day 28
Control	17	Male	5.0	15.9	11.9	18	14.5
Intervention	22	Male	4.2	16.7	12.2	17.9	18.7
Intervention	30	Female	6.2	15.5	11.2	16.3	17.2

## 4 Discussion

In this small study low‐dose primaquine (3.5 mg/kg) given over 7 days (0.5 mg/kg/day) appeared well tolerated, even in patients with intermediate (4–6 U/g Hb) G6PD activity and was not associated with any episodes of *P. vivax* parasitemia in patients initially presenting with either *P. falciparum* or *P. vivax* malaria. The only episode of *P. vivax* parasitemia within 6 months occurred in the control arm. The small sample size, the low number of recurrences and the absence of a comparator arm in which *P. vivax* patients were not treated with primaquine limit the interpretation of anti‐relapse efficacy. Furthermore, follow‐up was curtailed at 6 months and thus may have missed late‐latency relapses beyond this time point. However, an earlier study in Nepal reported that the risk of recurrence at 1 year was 4.1% in patients treated with chloroquine plus low‐dose primaquine over 14 days (0.25 mg/kg/day) compared to the background risk of 28.2% when no primaquine was provided [15]. In the same study, the risk of *P. vivax* at 6 months in the control arm was 18.6% with a cluster of patients presenting with homologous recurrences after 9 months [15].

Shorter primaquine regimens have the potential to improve effectiveness due to better adherence. The benefit of shortening the duration of treatment has been acknowledged by the WHO, which, in its 2022 guidelines revision, recommended the 7‐day low‐dose primaquine regimen as an effective alternative to the prolonged 14‐day course [4]. The more recent 2024 WHO recommendation advocates for a higher total dose of 7 mg/kg [8] based on global evidence of increased anti‐relapse efficacy [7]. However, in South Asia, the benefits of a high total dose (7 mg/kg) may be marginal compared to the standard 3.5 mg/kg regimen, suggesting that a low‐dose treatment might be sufficient in this region [8].

Shorter treatment regimens require a higher daily dose to achieve the same total dose, and this increases the risk of haemolysis in patients with low G6PD enzyme activity, thus emphasising the importance of G6PD testing. In this study, patient eligibility was assessed at the 30% enzyme activity level in line with current WHO recommendations for the 0.5 mg/kg daily doses of primaquine [8]. Almost half of the recruited patients had intermediate G6PD enzyme activities between 4 and 6 U/g Hb. While reassuring, the study was not powered to detect rare adverse events. Only one patient was excluded from the study due to G6PD deficiency (< 4 U/g Hb). The prevalence of G6PD deficiency in Nepal varies by region and between ethnic groups, ranging from nil to nearly 18%, with three main variants (Mahidol, Coimbra and Mediterranean) concentrated across the southern plain of the country [16]. While G6PD testing is not mandatory prior to primaquine treatment, it is encouraged in the Nepalese malaria treatment guidelines [17]. Implementation into routine care is however lacking mainly because of a lack of routine supply of testing equipment and regulatory supervision.

In this study G6PD activity was measured using the STANDARD G6PD, a semi‐quantitative test developed to guide tafenoquine treatment and identify patients with intermediate G6PD activities [12]. Providing treatment to patients at the 30% threshold would only require a qualitative test, expected to be cheaper and easier to use. However, no qualitative point of care test is currently WHO prequalified and widely available. The importance of qualitative testing to guide treatment in settings where 0.25–0.5 mg/kg daily doses are used has been raised previously [13].

Although the low number of malaria cases enrolled in this study reflects a decreasing burden of malaria in Nepal, safe and effective radical cure of *P. vivax* remains critical for achieving elimination targets. Conducting clinical trials in a pre‐elimination setting is inherently challenging, as declining case numbers limit recruitment and preclude large‐scale studies. A large proportion of malaria cases in Nepal are categorised as imported cases [1]. Mobile and migrant populations are highly relevant for malaria elimination efforts but are usually excluded from clinical studies because of concerns over incomplete follow‐up and inability to ascertain endpoints [18]. Nevertheless, even small, carefully documented trials can provide valuable operational insights and contribute to the evidence base needed to guide national policy and sustain elimination efforts. The decline of malaria cases in Nepal explains the slower than expected recruitment into this study, and the overall reduced recruitment numbers. National Malaria Program data suggest a 92% reduction in the overall malaria burden between 2002 and 2016 [19]. A similar trial conducted between 2015 and 2017 was able to recruit more than 200 vivax patients in 2 years [15]. More recent data from the National Malaria Program indicate a total of 1043 recorded cases in 2024, of which only 37 were classified as indigenous [1].

The reduced sample size and the small number of events precluded subgroup analyses by parasite species at enrolment. As a result, the potential benefit of universal radical cure, demonstrated in previous studies [12, 20] could not be assessed.

## 5 Conclusions

Despite its limitations, this small, randomised trial suggests that low‐dose primaquine (3.5 mg/kg) administered over 7 days (0.5 mg/kg/day) is well tolerated, including in individuals with intermediate G6PD activity (4–6 U/g Hb). The findings support the feasibility and safety of treatment with 7‐day low‐dose primaquine in Nepal, a regimen that offers potential programmatic advantages for improving adherence, enhancing effectiveness and facilitating the final stages of malaria elimination in the country.

## Funding

This work was supported by the Australian Academy of Science Regional Collaborations Program, Bill and Melinda Gates Foundation (OPP1164105/INV‐010504) and National Health and Medical Research Council (NHMRC) (GNT1132975). K.T., R.N.P. and J.A.S. are funded by NHMRC Leadership Investigator Grants (GNT2033264, 2008501 and 1196068). M.R. is partly funded through an NHMRC Synergy Grant (GNT2018654). This project was supported by the NHMRC Centre of Research Excellence (GNT2024622).

## Ethics Statement

The study protocol was reviewed and approved by the Human Research Ethics Committee of the Northern Territory Department of Health and Menzies School of Health Research (Reference number 2019‐3467) and the Nepal Health Research Council (Reference number 857/2019 P).

## Consent

Written informed consent was obtained from all participants prior to enrolment. The trial is registered with ClinicalTrials.gov (Identifier: NCT04079621).

## Conflicts of Interest

The authors declare no conflicts of interest.

## Data Availability

The database is closed and the data extracted and stored on WorldWide Antimalarial Resistance Network (WWARN.org) servers. De‐identified individual participant data are available following application to the WWARN Data Access Committee.
